# Simulation and Stability Assessment of Anti-EpCAM Immunotoxin for Cancer Therapy

**DOI:** 10.15171/apb.2018.052

**Published:** 2018-08-29

**Authors:** Seyed-Ali Hosseinian, Aliakbar Haddad-Mashadrizeh, Samaneh Dolatabadi

**Affiliations:** ^1^Department of Biology, Khorasan Razavi Science and Research Branch, Islamic Azad University, Neyshabur, Iran.; ^2^Department of Biology, Neyshabur Branch, Islamic Azad University, Neyshabur, Iran.; ^3^Cell and Molecular Biotechnology Research Group, Institute of Biotechnology, and Department of Biology, Faculty of Science, Ferdowsi University of Mashhad, Mashhad, Iran.

**Keywords:** EpCAM, Cancer therapy, Immunotoxin, Simulation, Stability

## Abstract

***Purpose:*** Epithelial cell adhesion molecule (EpCAM) is a dominant antigen in human colon carcinoma tissue. Topology features of this antigen are different in normal and malignant conditions; for instance, EpCAM is much less accessible to antibodies in normal cells than in cancerous tissues. Hence, EpCAM has been considered as a suitable candidate for cancer target therapy via immunotoxins (ITs) development. In this study, attention was focused on the stability assessment of anti-EpCAM-IT (anti-Ep-IT) to design a novel IT.

***Methods:*** The 3D structures of the antibody template and the toxin segment of anti-Ep-IT were retrieved from PDB. Discovery Studio3.0 was used to separate the ligands and water molecules. The antibody (Ab) fragment of anti-Ep-IT was aligned using protein blast (BLAST-p), and SWISS-MODEL database was used for Ab modeling. IT modeling was accomplished using MODELLER 9.15. Also, GROMACS 5.07 was used for molecular dynamic (MD) simulation step. Moreover, ERRAT and RAMPAGE databases were used for quality assessment of the structures.

***Results:*** BLAST-p results indicated that antibody moiety of IT has the highest E-value and query coverage scores to the monoclonal antibody (mAb) 4D5MOC-B. Modeling by SWISS-MODEL provided a reasonable template for Ab portion compared to MODELLER. The best modeled full-length IT with the lowest RMSD values was selected. Finally, RMSD plot for MD stage demonstrated constant values from 7000ps to 20000ps.

***Conclusion:*** In general, both modeling results and their quality evaluations were satisfactory for designing IT. Moreover, RMSD plot revealed that IT stability was preserved during the simulation. Overall, our findings led to modeling and simulation of the anti-Ep-IT with more structural stability.

## Introduction

Originally discovered in 1979, epithelial cell–adhesion molecule (EpCAM, CD326), type I, transmembrane, 39–42 kDa glycoprotein, was first described as a dominant antigen in human colon carcinoma.^1^ EpCAM is frequently expressed in human epithelial tumors, mainly in adenocarcinomas and other carcinomas, and it is also stably expressed and even upregulated during the progression of cancer.^2^ The role of EpCAM is not limited to cell adhesion; it is also involved in cellular signaling, cell migration, proliferation, and differentiation.^3,4^ EpCAM can be used as a prognostic marker. In certain types of tumors, EpCAM is associated with advance stage disease and overall survival.^5^ The association of EpCAM with proliferation, adhesiveness, tissue stabilization, promotion of tumor growth, and metastasis suggests that EpCAM is a pleiotropic molecule that potentially offers therapeutic applications in cancer treatment.^6^ Generally, the expression of EpCAM is restricted to epithelia. More specifically, EpCAM is detected in the basolateral cell membrane of normal human tissue.^7^ Moreover, EpCAM is believed to be an early marker for (pre)malignancies.^8^ Enhanced EpCAM expression was found in preneoplastic epithelial of the colon. In colorectal, pancreas, bladder, prostate, breast, and ovarian carcinoma, EpCAM was more intensely positive in tumor cells than in the corresponding normal tissues.^9^ Ep-CAM-specific antibodies have been used to image and detect primary tumors and metastases in patients with small cell lung cancer and non-small cell lung cancer. Among anti-Ep-CAM MAbs, PANOREX®, which is a murine monoclonal antibody, also known as edrecolomab, has been approved to be used in the treatment of colon cancer in Germany.^10,11^ PANOREX® treatment has been associated with undesirable side effects, including abdominal cramps, nausea, transient diarrhea, and cutaneous urticarial lesions.^12-14^ However, the search for an effective, low-toxicity, anti-Ep-CAM antibody continues.^15^ A humanized, stabilized, single-chain, anti-Ep-CAM antibody, 4D5MOC-B, which is derived from murine monoclonal antibody MOC31, has also been developed.^16^ Immunotoxins (ITs) are therapeutic agents with a high degree of specificity and unique mechanism of action. An immunotoxin is a chimeric protein that consists of a targeting moiety linked to a toxin. The targeting moiety selectively binds to a tumor cell and targets it for death via the attached toxin. Generally, immunotoxins are specifically potent against cancer. However, immunotoxins can be limited clinically by immunogenicity, toxicity, and instability.^17^ With the advance of recombinant DNA technology, the second-generation ITs are entirely recombinant in the sense that the gene for the Fab or Fv fragments of an antibody is fused with the gene encoding a truncated toxin, followed by transfecting the plasmid vector expressing the recombinant IT into bacteria or yeast for single-chain fusion protein production.^18,19^

The Fv fragment of an antibody is a heterodimer consisting of heavy-chain (V_H_) and light-chain (V_L_) domains and is the smallest functional module retaining the full antigen-binding specificity.^20^ To enhance the efficacy of these recombinant immunotoxins (RITs) in treating malignant tumors, great effort has been devoted to reduce the RITs size, identify better antigen targets, improve RIT binding affinity and stability,^21,22^ prolong retention in the circulation,^23,24^ reduce immunogenicity,^25,26^ or explore the intracellular translocation pathway for RIT fragments.^27^

## Materials and Methods

To use the toxin subunit in full-length immunotoxin modeling, the crystal structure of the wild-type of Pseudomonas aeruginosa exotoxin A (ETA) (with PDB ID: 1IKQ and 1.62 Å resolution), as the initial structure for the modeling and simulations procedures, was taken from the Protein Data Bank (PDB). To eliminate useless moiety of ETA and the ligand and water molecules from native structures, Discovery Studio Client 3.0‏ was used; then, new structures were saved again as pdb format. The protein sequence of anti-Ep-IT was obtained from online Google patents. In the next stage, fasta format file of antibody (Ab) moiety was generated. Ab sequence was aligned using BLAST-p program. Subsequently, pattern Ab subunit was retrieved from PDB (with PDB ID: 3AUV and 2.4 Å resolution). MODELLER9.15 software and SWISS-MODEL online program were used to model Ab subunit. Full-length IT was generated by MODELLER 9.15 with multiple align command file. The energy minimization of the globular structure of IT was accomplished with MOE program. The MD simulation was performed by GROMACS 5.07 software with a GROMOS96 43a1 force field. The well-tested SPCE model was used for water molecules, and water molecules in the crystal were reserved during the simulation. The protein was solvated in a cubic box, and the box size was chosen by the following criterion: the distance of protein atoms from the wall had to be greater than 1 nm. The system equilibration was performed gradually at 313ºK for 100ps of simulation time in an NVT ensemble and 100ps simulation at 313ºK for data sampling in an NPT ensemble. The last step of simulation was MD run at 313ºK for 20000ps (20ns) of simulation time. Visualizing the secondary structures of modeled and simulated proteins were accomplished via Pymol. Finally, the output files of MD stage were used for generation of momentary, average pdb files, and RMSD data file. In addition, to draw RMSD plot, Excel was used. Eventually, responsible online programs exhibited Ramachandran and ERRAT plots.

## Results

### Identifying IT components

To explore structural and functional regions, the protein sequence of IT was investigated. The nucleotide and polypeptide sequences can be divided into the following domains: the signal sequence for periplasmic expression; histidine tags; Ab complementarity-determining regions (CDR) 1, 2, and 3 domains; V_L_ domain; V_H_ domain; linkers; ETA domains II, Ib, III; and an endoplasmic reticulum (ER) retention signal KDEL. Ab fragment is made of the single-chain variable fragment of the‏ monoclonal antibody (mAb) 4D5MOC-B, which is a humanized single-chain variable-fragment (scFv) MOC31 antibody and is composed of V_L_ and V_H_ chains. The toxin portion consists of 252 to 608 amino acids (aa) of native ETA. The centrally located domain II is small and includes residues 253-364 and is composed of 6 alpha helices. In the linear amino acid sequence, domain II separates domain I into 2 subdomains: (1) domain la (aa 1-252) and (2) Ib (aa 365-404). The crystalline structure reveals that 3 of the disulfide bonds of ETA are within domain I and the fourth is in domain II. Domain III, the enzymatic domain encompassing the carboxy-terminal residues 405-613, exhibits a less regular secondary structure than the other 2 domains. An interesting feature of the model proposed for ETA is an extended cleft in domain III, and the active site of the enzyme is within this cleft.

### Modeling IT components

For full-length IT assemble, at first, it is necessary to build Ab and toxin models and save them as pdb format as major input files. In this regard, crystallography structure of ETA was obtained and then the IA domain of ETA was eliminated from the whole toxin structure to gain toxin model. For model Ab structure outset, Ab was aligned using BLAST-p. The results revealed that a single chain disulfide-stabilized antibody variable fragment (sc-dsFv), derived from the G6-Fab (with PDB ID: 3AUV), had the most similarity to the query sequence (4D5MOC-B mAb). This mAb (G6) consists of 6 chains, and residues of chain A (3AUV_A) has the most sequence coverage to 4D5MOC-B mAb in the region of 1-252. The BLAST scores were as follow: E-value = 9e-106, query coverage = 99%, and max identity = 66%. Thus, 3AUV_A pdb file was used as the template structure for Ab modeling. Subsequently, the display of secondary structures using Pymol software indicated that in the model developed by the MODELLER software, the VL-VH linker (in the position of 142-165 amino acids) changed to beta structure and 27 N-terminal region amino acids changed to coil, and thus it did not conform to the usual conventional observation of antibodies. Accordingly, modeling Ab moiety again was performed via SWISS-MODEL. In the obtained model, V_L_-V_H_ linker sequence was located within the coil protein construction and both Ab chains (V_L_ and V_H_) were also displayed as globular structures (normal secondary structures).

### Modeling full-length IT

First, the template and input files for modeling based on protein sequences of Ab and ETA subunits were defined. The arrangement of all IT subunits was in “align” file and was exactly compatible with protein sequence of the whole IT. At the end (modeling), among the 10 possible structures, the best structure was selected qualitatively. For the initial verification of IT modeling, the favored structure was compared with the reported pattern in the reference patent document.

### Molecular **simulation** of IT

**Figure 1:**
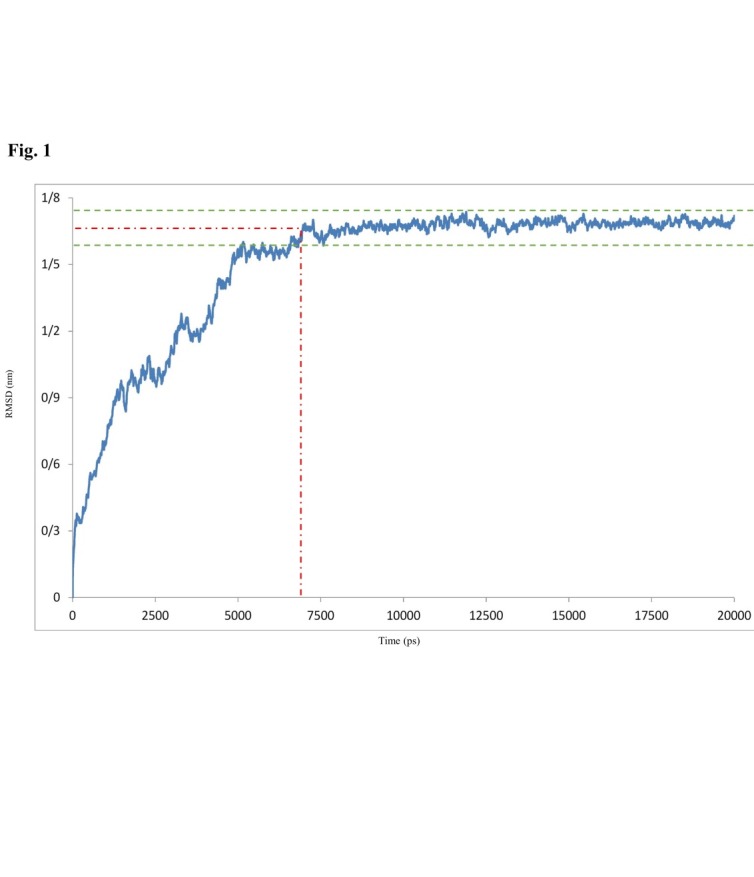
RMSD of the Cα atoms of overall conformation of simulated IT in 20000ps of simulation time

With the aim of stability evaluation of protein construction of modeled IT in an explicit water solvent environment, MD simulation was used throughout GROMACS. To assess conformational stability rate during the simulation, RMSD of the Cα carbon for overall IT structure in the physiological condition of the body (including pH = 7, temperature = 37ºC or 313ºK and aqueous solution) was computed. As shown in Figure 1, RMSD values had an increasing trend, ranging from 0ps to 7000ps, and then they remained roughly constant to the end of the simulation (20000ps). In other words, from 7000ps to 20000ps, RMSD values fluctuate between about 1.53Å to 1.72Å. Hence, the instantaneous configuration of the structure was created for these 3 times (0ps, 7000ps, and 20000ps). To evaluate stability in the obtained conformations, changes in secondary structures were scrupulously compared together.

### Assessment of Conformational changes

**Figure 2:**
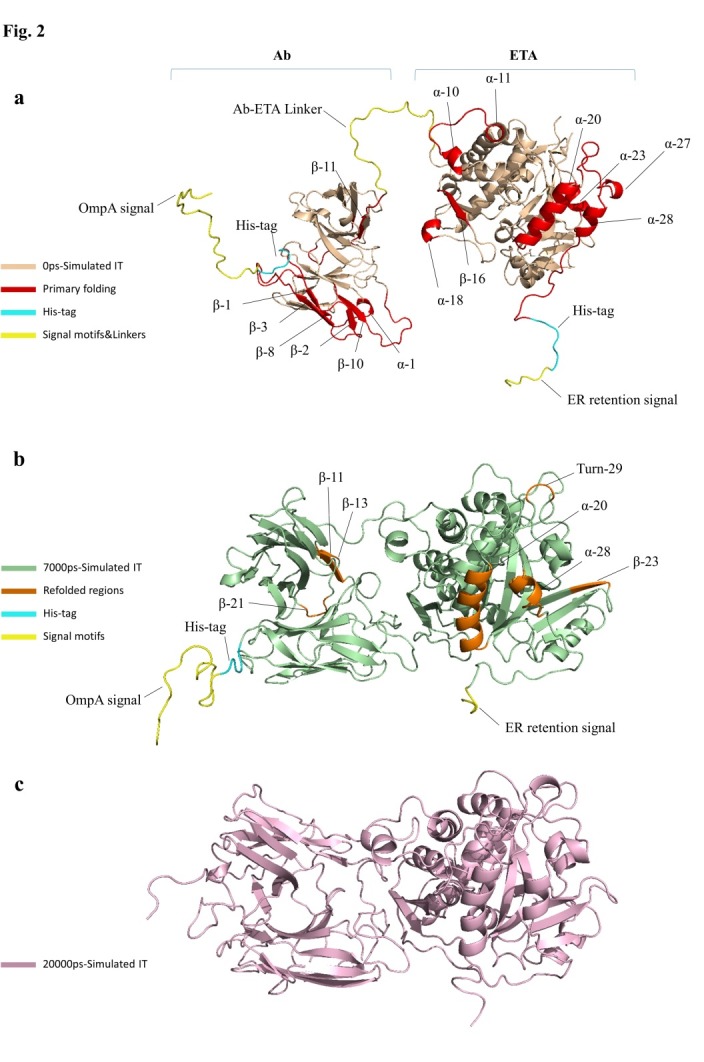
Shifting conformational stability of IT secondary structures in (a) 0ps, (b) 7000ps and (c) 20000ps of simulation time

The visualization of conformations in the aforementioned times specifically indicated that at the beginning of the simulation procedure (Figure 2a), compared with the start point of equilibrium (7000ps) (Figure 2b), some conformations had high fluctuation of basic state or they even shifted to other forms of secondary structures. At first glance, of these undulations, the highest fluctuations were associated with special coil conformations, with having 1 free terminal (such as initial OmpA signal peptide and terminal ER retention signal peptide) as well as long sequence coils (such as V_L_-V_H_ linker and Ab-ETA linker). As expected, structures that did not have a secondary structural pattern (such as the His-tag motif) changed to coil. Moreover, as demonstrated in Figure 2a, 3 cores of fluctuation have been distinguished in overall conformation in the initial moment, which are as follow:

(1) the initial part of the subunit antibody chain (including beta 1, 2, 3, 8, 10, and alpha 1 spirals) adjacent to the VL-VH linker; (2) some parts of the dermal II (including alpha 10, 11, 16) and dominant Ib (including Helix 3.10 18) exotoxin A; (3) Dimension 3 (including Alpha 20, 23, 27, 28) exotoxin A.

In addition, some shifting was distinctive in particular spaces, such as beta strand 11 of Ab in VH domain. Further, more exploration of shifting conformation in 7000ps moment, compared with the end point of MD simulation (20000ps) (Figure 2b), revealed that although overall conformation progressed toward more stability, some of the above-mentioned regions (such as beta strand 11 in Ab moiety and alpha helixes 20, 28) still had some fluctuations. Also, there were new regions that shifted to other conformations. These points comprised of beta strands 13 and 21 in Ab section and beta strand 23 and turn 29 in ETA section, all of which having shifted to the coil conformation (Figure 2b). At the end of the procedure (20000ps) (Figure 2c), overall conformation of IT took a globular form (general form of the secondary structure of protein). Also, both subtypes of antibodies and exotoxin A were aligned. On the other hand, the regions that did not have any reasonable pattern for their secondary structures, such as initial and terminal signal peptides and linkers, were more folded and thus assumed more stability (Figure 2c).

### Conformations alignment

**Figure 3:**
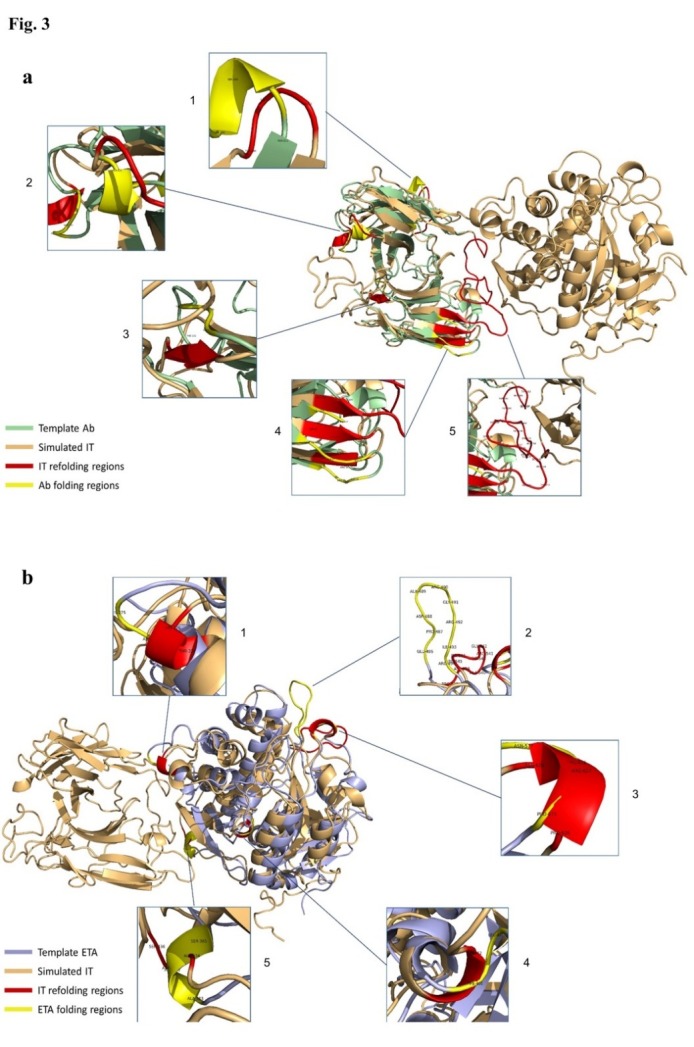
Alignment of Ab (a) and ETA (b) fragment for simulated IT

**Figure 4:**
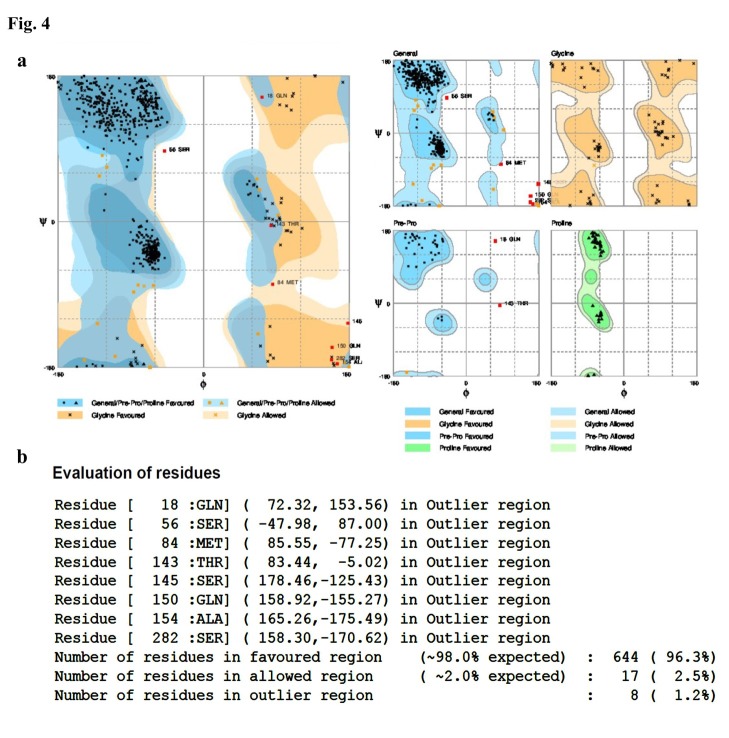
Ramachandran plot of simulated IT (a), and evaluation of its residues (b)

To verify the conformational authenticity of the final simulated IT, alignment of Ab and ETA fragments to the templates conformations was accomplished. The achieved results for the alignment of Ab subunit of simulated IT to template Ab, with PDB ID: 3AUV-A (Figure 3a), clearly led to identifying 8 distinguished refolded regions compared with crystallography structure of Ab template. Of these conformation changes, 2 coil to turn shifting occurred, one segment in Ile163, Ser164, Asp165 residues (Figure 3a-1), and another in Ala254, Glu255, Asp256 residues (Figure 3a-2), respectively. The one coil to turn shifting was situated in Asp228, Ser229, and Phe230 residues (Figure 3a-2). In addition, 4 coil to beta change occurred, with one in Thr130, Phe131, and Gly132 residues (Figure 3a-3) and 3 others in Ser39, Ala40, Ser41, Asp45, Arg46, Val47 residues, and Glu138, Leu139, and Lys140 residues (Figure 3a-4), respectively. Eventually, the final conformational change corresponded to V_L_-V_H_ linker motif of Ab part of IT. In fact, this section was not in Ab template. As demonstrated in Figure 3a-5, because of lack of templates for linker secondary structure, coil conformation may be more appropriate. On the other hand, in the alignment of ETA subunit of IT to the equivalent crystallography structure of ETA with PDB ID (1IKQ; Figure 3b), there were 4 regions with different configurations plus a change of direction compared to the original natural structure. Among these 4 accurate conformational shifting, there were 3 coil to helix shifting in the locations of Thr324, Arg325, and His326 residues (Figure 3b-1), Pro626, Arg627, Asn628 residues (Figure 3b-3), and Phe451and Leu452 residues (Figure 3b-4). Moreover, 1 helix to coil shifting occurred in Ala434, Asp435, and Ser436 residues (Figure 3b-5). Furthermore, a direction shifting occurred at the loop conformation in the location of Glu537, Pro538, Asp539, Ala540, Arg541, Gly542, Arg543, Ile544, and Arg545 residues (Figure 3b-2) in the overall IT sequence.

### Structure quality assessment

To evaluate the quality of modeled Ab (4D5MOC-B) alone, and overall modeled and simulated ITs, Ramachandran plots were created, which showed the distribution of *φ* and *ψ* angles in the modeled and simulated models‏. In association with Ramachandran plot of Ab part alone, of 252 residues existing in sequence, 88.5% of residues (223 aa) were located in the most favored region, 9.1% (23 aa) in the allowed region, and only 2.4% (6 aa) in the outlier region (data not shown). In addition, plots were created for both modeled and simulated conformations of IT. According to the plot statistics, as shown in Figure 4a, of the total number of 669 residues of overall IT sequence, 96.3% of residues (644 aa) were located in the most favored region, 2.5% (17 aa) in the allowed region, and only 1.2% (8 aa) in the outlier region, despite intensity of residues number. As displayed in Figure 4b, the 8t residues that were located in the outlier region comprised of Gln18, Ser56, Met84, Thr143, Ser145, Gln150, Ala154, and Ser282 residues. Among these residues, Gln18 was in the position of OmpA initial signal and Ser56 and Met84 were in V_L_ chain of Ab fragment. Furthermore, Thr143, Ser145, Gln150, and Ala154 residues were located in V_L_-V_H_ linker, and Ser282 in V_H_ domain. The quality protein structure was verified with ERRAT, where ERRAT plot showed overall quality factor 79.668 for 4D5MOC-B. These values for the completely modeled and simulated ITs were 62.323 and 80.514, respectively (data not shown).

## Discussion

Functional characterization of a protein sequence is one of the most frequent problems in biology. In the absence of an experimentally determined structure comparative or homology modeling can provide a useful 3D model for a protein (target) that is related to at least one known protein structure (template).^28-30^ Even models with errors may be useful because some aspects of the function can be predicted only from coarse structural features of a model.^30^ In the first stage of this study, the alignment of the query Ab (4D5MOC-B) to template Ab (3AUV-A) represented 99% query coverage and 66% identity value. Modeling structures in all patterns associated with models, based on 50% sequence matching or more, generally have no errors.^31^ Models with such high accuracy have been shown to be useful even for refining crystallographic structures by molecular replacement method.^32^ Nevertheless, the resulting conformation of MODELLER program, due to unfolding N-terminal region of Ab V_L_ domain as well as beta folding V_L_-V_H_ linker instead of the common coil form, was ineligible. Therefore, in the modeling process, we used SWISS-MODEL that led to creating the compatible model with known Ab conformations for crystallography structures (template). Ramachandran plot criteria for backbone conformation emphasize on distinguishing the rare from erroneous *φ*, *ψ* values, touched briefly on sidechain criteria.^33,34^ Ideal bond angle values are known through highly accurate small molecule structures^35^ and traditional structure validation reports^36,37^ flag outliers that deviate by more than a few standard deviations.^32^ A residue with the good fit to favored *φ*, *ψ* values, no atomic clashes, and ideal covalent geometry is almost certainly modeled correctly.^32^ Thus, among 10 modeled conformations via MODELLER in both Ramachandran and ERRAT criteria, the best model with almost 98.8% of residues located in appropriate position, ERRAT score 62.323, is preferred. The obtained structure of overall IT simulation in physiologic conditions (313ºK, pH = 7) showed that the ERRAT overall quality factor increased to 80.514, representing more stable conformation during the simulation procedure. Moreover, it is deduced from the RMSD chart that after only 7000ps of simulation time due to reaching (RMSD values) to a constant mean value of about 1.6 Å, the configuration was further improved to a greater stability. Comparison of RMSD and conformations results at specified intervals revealed that the high fluctuation of RMSD value in the initial moment of simulation caused instability of special sections of MD simulated IT. These regions comprised of conformation without pattern motifs, such as signal peptides and linkers as well as some secondary structures containing most of the beta sheets of Ab V_L_ chain together with several regions of alpha helices form of II, Ib, and III domains of ETA. Corresponding to 7000ps momentary conformation, there were fewer fluctuating and refolding regions, including 3 beta strands in V_H_ chain of Ab and 2 alpha helices, 1 beta strand, and 1 turn folding, which were completely located in the catalytic domain of ETA (domain III). Then, the structure fluctuations were reduced in the interval from 7000ps to 20000ps because of the stability of the RMSD values. Overall, at the end of the simulation (20000ps), the comprehensive structure of IT was directed to folded conformations for both Ab and ETA moieties. To confirm the authentication of MD simulation, Ab and ETA subunits of the average simulated IT conformation were aligned with native and template structures. In this regard, the Ab alignment exhibited 8 refolding (7 refolded regions+1 excess V_L_-V_H_ linker) that included 4 coil→beta, 1 coil→turn, 2 turn→coil, and 1 linker in the form of coil. Considering that beta and turn folding had more stability compared to coil folding, because of interchain hydrogen bonds, 5 of 8 refolding (4 coil→beta+1 coil→turn) led to creating more stable conformation. Generally, the folding linker VL-VH should be in the form of coil. The most important challenge of the mentioned IT simulation was placing 2 remaining refolded parts (turn→coil) in 240-242 and 254-256 regions of IT sequence. As these regions were precisely located in the position of antigen binding site of V_H_ chain of Ab (*paratope areas)*, they could interfere with Ab-antigen binding procedure. In addition, the turn-to-coil transformation is a source of instability in the immunotoxin conformation due to the reduction in the number of hydrogen bonding in the globular structure, so it could partly cause instability of simulated conformation. ETA fragment of overall IT and its native 3D structure were aligned in the last stage of conformational comparison. The results revealed that despite using the crystallographic structure of ETA with PDB ID (1IKQ as toxin moiety template of IT in energy minimization step of MD simulation), ETA structure developed to more stable and refolded conformation. In this regard, 4 refolded regions of 5 prominent refolding have progressed into the more established configuration that contained 3 coil→helix refold plus a loop folding of 538-545 residues of comprehensive IT. It seems that changes occurred due to elimination of Ia domain from the native structure and the combination of unfolded motifs of Ab-ETA linker and ER retention signal peptide to N- and C-terminal domains of ETA. It is considered that of 5 rearrangements of ETA fraction, 1 territory was correlated to domain II, 2 to domain Ib, and 2 others corresponded to domain III or catalytic domain of ETA. Finally, the overall outcomes of the quality assessment based on Ramachandran plot and ERRAT characterization revealed a higher quality of simulated IT conformation, with 98.8% residues located in the authorized area and 80.514 for overall quality factor, compared to early modeled IT and Ab fragment alone.

## Conclusion

Recent advances in antibody engineering have provided a number of functionally optimized anti-EpCAM antibodies.^38^ Anti-EpCAM antibody conjugated with alpha-amanitin has the potential to be a highly effective therapeutic agent for pancreatic carcinoma and various EpCAM-expressing malignancies.^39^ Since 1970s, the method of MD simulation has gained popularity in biochemistry and biophysics.^40^ The aim of this study was to offer a reasonable pattern of anti-EpCAM IT model to design the novel generation of recombinant IT. Determining stability of IT and identifying unstable regions can lead us to increase efficiency and sustainability of anticancer drugs in physiologic conditions. Overall, our findings led to validation of the simulated stability model for intended IT in most areas.

## Acknowledgments

Authors are thankful to the Islamic Azad University of Neyshabur and gratefully acknowledge Khayyam bioeconomic company of Mashhad for allowing to use all computational facilities.

## Ethical Issues

Not applicable.

## Conflict of Interest

The authors declare no conflict of interests.
